# Database-Guided Local Refinement and Truncated Power Estimation for LEO Uplink Anti-Interference Beamforming

**DOI:** 10.3390/s26113482

**Published:** 2026-06-01

**Authors:** Jian Zhang, Xuekai Zhang, Tianqi Sheng, Zhiyong Lv, Chao Yuan, Zhou Zhou, Peng Chen

**Affiliations:** 1 State Grid Shandong Electric Power Company, Jinan 250001, China; 2 State Grid Yantai Power Supply Company, Yantai 264000, China; 3 State Grid Laiwu Power Supply Company, Laiwu 271100, China; 4 State Key Laboratory of Millimeter Waves, Southeast University, Nanjing 210096, China

**Keywords:** adaptive beamforming, database-assisted, interference suppression, LEO satellites, satellite communication

## Abstract

Uplink emergency communications for LEO satellites are vulnerable to severe co-channel interference from dense terrestrial networks. This paper proposes a database-aided robust adaptive beamforming framework that treats database angles as coarse priors and compensates for spatial mismatches. The method includes three stages: (1) local spatial refinement via constrained Capon search to accurately locate interferers while protecting the desired signal; (2) truncated adaptive amplitude (TAA) estimation to recover true interference powers and suppress noise artifacts; and (3) reconstruction of the interference-plus-noise covariance matrix (INCM) for MVDR beamforming. By avoiding global angular scanning and improving spatial statistics estimation, the approach achieves near-optimal SINR under limited snapshots and strong interference. Simulations show consistent performance gains over DL-MVDR, ESB, IAA, and existing database-assisted methods across varying SNR, INR, and mismatch conditions, demonstrating strong robustness and practical applicability.

## 1. Introduction

Reliable communication is a fundamental requirement for emergency response and disaster recovery operations, where timely information exchange is critical for coordination, situational awareness, and public safety [1]. For large-scale infrastructure operators such as power grid corporations, petroleum enterprises, and transportation networks, communication outages during extreme events may severely hinder rescue operations and system restoration. Unfortunately, terrestrial communication infrastructures are often highly vulnerable to natural disasters: earthquakes can sever optical fibers, floods can disable base stations, and severe weather can disrupt cellular coverage precisely when communication demand peaks.

Satellite communication systems provide an indispensable backup solution for such emergency scenarios due to their wide coverage, rapid deployment capability, and independence from terrestrial infrastructure [2]. In particular, low Earth orbit (LEO) satellite constellations have attracted increasing attention owing to their reduced propagation delay, lower path loss, and improved link budget compared with traditional geostationary Earth orbit (GEO) satellites [3]. As a result, LEO satellite communication has been actively incorporated into emergency communication frameworks to support real-time monitoring, remote control, and coordinated command during large-scale disasters [4].

Despite these inherent advantages, the uplink of LEO satellite systems faces a critical and escalating challenge: severe aggregate interference from dense terrestrial wireless networks [5]. With the rapid deployment of fifth-generation (5G) and beyond cellular systems, satellite–terrestrial spectrum sharing in sub-6 GHz and millimeter-wave bands has become increasingly common to alleviate spectrum scarcity [6,7]. Although such sharing paradigms significantly improve overall spectral efficiency, they inevitably introduce strong co-channel or adjacent-channel interference to satellite uplinks [8]. This problem is uniquely exacerbated in LEO systems due to their orbital characteristics [9]. A single LEO satellite footprint typically spans hundreds to thousands of kilometers, potentially covering tens of thousands of active terrestrial transmitters simultaneously [10]. Because of the high probability of line-of-sight (LoS) propagation between ground base stations and the satellite, the aggregate interference power received at the satellite antenna array can easily exceed the weak desired emergency uplink signal by tens of decibels. This overwhelming interference floor severely degrades the signal-to-interference-plus-noise ratio (SINR), leading to unacceptable packet loss and potentially rendering critical emergency communication links entirely unusable.

Adaptive beamforming at the satellite receiver is widely recognized as one of the most effective techniques for mitigating such uplink interference [11]. By exploiting the spatial degrees of freedom provided by multi-element antenna arrays, adaptive beamforming can synthetically enhance the antenna gain toward the desired signal while placing deep spatial nulls toward the interference directions. Among existing spatial filtering methods, the minimum variance distortionless response (MVDR) or Capon beamformer is theoretically optimal in maximizing the output SINR, provided that the interference-plus-noise covariance matrix (INCM) and the desired signal steering vector are perfectly known [12]. However, the practical implementation of conventional MVDR beamforming in emergency LEO scenarios is severely hindered by several fundamental limitations. First, accurate estimation of the INCM typically requires a large number of stationary data snapshots. In fast-changing emergency conditions with high-mobility LEO satellites, the channel coherence time is extremely short, resulting in a severely snapshot-limited regime where the sample covariance matrix (SCM) becomes ill-conditioned [13]. Second, the desired signal component inevitably contaminates the SCM, leading to the well-known signal self-nulling phenomenon and drastic performance loss [14]. Third, steering-vector uncertainties caused by array calibration errors, thermal deformation, and satellite attitude jitter further degrade the beamformer’s robustness [15,16].

To address these issues, extensive research has been devoted to robust adaptive beamforming (RAB). Diagonal loading MVDR (DL-MVDR) enhances robustness by adding a scaled identity matrix to the SCM, effectively penalizing large weight norms [17]. Robust Capon beamforming (RCB) further formulates steering-vector uncertainty using ellipsoidal constraint sets, providing a principled mathematical approach to determine the optimal loading factor [18]. Recent studies have also explored data-driven strategies to automate diagonal loading selection, such as utilizing deep neural networks to estimate the signal-plus-interference subspace dimension [19,20]. Although these methods reduce manual parameter tuning, their performance remains fundamentally bottlenecked by the quality of the initial SCM estimation, making them highly vulnerable in snapshot-starved environments [21].

Eigenspace-based beamforming (ESB) represents another important class of robust techniques, where the nominal steering vector is projected onto an estimated signal subspace to prevent desired-signal cancellation [22,23]. Improved ESB variants have been proposed to enhance performance at high signal-to-noise ratios (SNR) while reducing computational complexity [24]. Nevertheless, ESB methods heavily rely on the accurate eigenvalue decomposition and subspace separation of the SCM. In low-SNR or snapshot-limited regimes, the signal and noise subspaces inevitably leak into each other, rendering the projection unreliable. Furthermore, these purely data-driven methods do not explicitly exploit any external prior information about the interference environment.

An alternative and highly effective line of work focuses on reconstructing the INCM using spatial power spectrum estimation, thereby completely eliminating the desired signal component from the covariance matrix. The power spectral estimation with uncertainty regions (PSEUR) method identifies interference sectors and reconstructs the INCM without requiring full-spectrum estimation [25]. The iterative adaptive approach (IAA) based beamforming reconstructs the covariance matrix from an iteratively refined spatial spectrum, demonstrating excellent resolution and the ability to handle coherent interference effectively [26,27,28]. However, both PSEUR and IAA typically rely on dense global angular grids covering the entire spatial domain. For a planar array on a satellite, a 2D global grid search incurs prohibitive computational complexity. Given the strict power and processing constraints of onboard satellite payloads, such computationally intensive global search methods are often impractical for real-time emergency deployments [29].

Recently, the exploitation of satellite-specific prior information has emerged as a promising paradigm to enhance interference mitigation without relying solely on limited snapshot data. Angular reciprocity-based methods [30] and radio environment map (REM)-assisted cognitive beamforming [31] leverage geometric or historical environmental knowledge to improve beamforming robustness. In particular, database- or location-assisted beamforming has gained traction in satellite–terrestrial coexistence scenarios. By querying a centralized spectrum database containing the geographical coordinates of registered terrestrial base stations, the satellite can obtain prior knowledge of potential interference directions. For instance, deterministic null placement based on terrestrial transmitter locations is studied in [32], while robust transmit beamforming using statistical channel state information (CSI) is considered in [33,34]. However, most existing database-assisted methods operate under the idealized assumption of perfectly accurate prior information, or they focus primarily on transmit-side interference constraints to protect ground users. They rarely address the critical issue of database uncertainty in receiver-side adaptive interference suppression.

In practical satellite–terrestrial systems, the angular information derived from databases inevitably contains non-negligible errors. These inaccuracies stem from multiple sources, including satellite ephemeris uncertainty, imperfect ground station location registration, coordinate transformation quantization errors, and dynamic satellite attitude jitter. Under strong interference conditions, even a fraction of a degree of angular mismatch can cause the spatial null to miss the true interferer, leading to catastrophic interference leakage and severe SINR degradation. When such mismatch is ignored, the performance of conventional database-assisted beamforming can be worse than blind data-driven methods. To the best of our knowledge, a robust receiver-side beamforming framework that can jointly exploit coarse database priors, explicitly compensate for angular mismatch, accurately reconstruct the INCM, and simultaneously avoid the computational burden of exhaustive global spatial searches remains insufficiently studied in the literature.

Motivated by these critical challenges, this paper proposes a novel database-aided robust adaptive beamforming framework specifically tailored for uplink interference suppression in LEO satellite emergency communications. Recognizing the unreliability of purely data-driven methods under snapshot starvation and the fragility of conventional database methods under angular mismatch, the proposed approach strategically bridges the gap between the two. Instead of treating database information as exact deterministic knowledge, the proposed framework models it as coarse prior guidance. It explicitly compensates for database errors through a localized spatial refinement mechanism and an adaptive power reconstruction process.

It is worth emphasizing that the proposed framework is fundamentally different from existing robust beamforming methods in three aspects. First, unlike conventional sample-covariance-based robust beamformers, the proposed method does not rely solely on the received snapshots to estimate the interference-plus-noise covariance matrix, which improves robustness under limited snapshot support. Second, unlike IAA-based methods that usually require global angular spectrum estimation, the proposed method exploits database information to restrict the refinement to local angular regions, thereby reducing the search dimensionality and computational burden. Third, unlike conventional REM/database-assisted beamforming methods that directly use nominal database directions, the proposed method explicitly compensates for database angular mismatch through local refinement and truncated adaptive amplitude estimation. Therefore, the proposed framework jointly integrates database guidance, mismatch-aware refinement, and robust covariance reconstruction into a unified beamforming architecture.

The main contributions of this work are summarized as follows.

- A database-guided local spatial refinement strategy is proposed, where database angular information is modeled as an uncertainty-bounded prior and refined by local Capon spectrum search, enabling accurate interferer localization under database mismatch.
- A truncated adaptive amplitude (TAA) estimation algorithm is developed to recover interference powers exclusively at refined database-guided directions, avoiding computationally expensive global spectral searches while suppressing spurious sources.
- Based on refined directions and power estimates, a precise INCM reconstruction method is formulated, yielding robust MVDR beamforming performance that approaches the theoretical optimum under strong interference and snapshot-limited conditions.
- Extensive simulations demonstrate that the proposed method consistently outperforms DL-MVDR, ESB, IAA, and a baseline robust database method across a wide range of SNR, interference-to-noise ratio (INR), snapshot, and database error scenarios.

Notations: Upper-case and lower-case boldface letters denote matrices and vectors, respectively. ${\left(\;\cdot \;\right)}^{T}$ and ${\left(\;\cdot \;\right)}^{H}$ denote transpose and Hermitian transpose. $I_{N}$ is the N×N identity matrix. E{·} denotes expectation. For a vector p, diag(p) denotes the diagonal matrix whose diagonal entries are given by p. ${∥\;\cdot \;∥}_{2}$ denotes the Euclidean norm, and $C^{N\times K}$ denotes the set of complex-valued N×K matrices.

## 2. System Model

**Figure 1:**
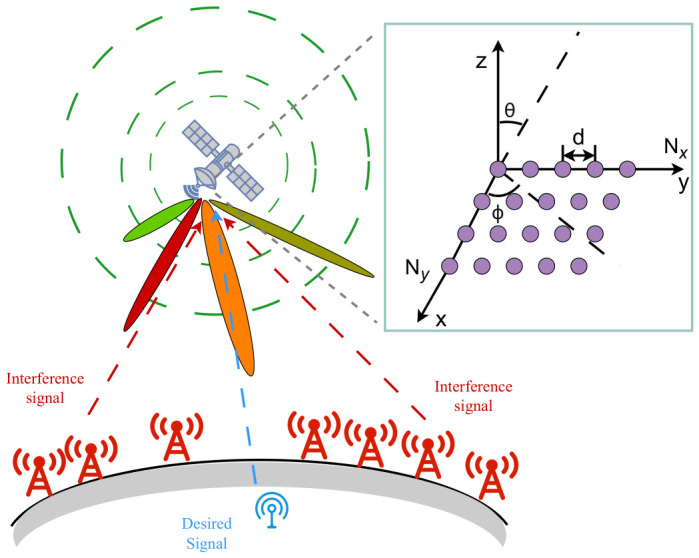
System architecture of the considered LEO emergency uplink with a satellite UPA receiver and dense terrestrial interferers.

Consider a LEO satellite uplink equipped with a uniform planar array (UPA) of size $N_{x}\times N_{y}$, where the total number of antenna elements is $N=N_{x}N_{y}$, as illustrated in Figure 1. The array lies on the xy-plane with an inter-element spacing d=λ/2, where λ is the carrier wavelength. Let θ∈[0°,90°] denote the elevation angle measured from the array normal (i.e., θ=0° corresponds to the broadside or zenith direction), and let ϕ∈[0°,360°) denote the azimuth angle measured counterclockwise from the positive *x*-axis.

Under the far-field narrowband assumption, the spatial steering vector for a signal arriving from direction (θ,ϕ) is given by the Kronecker product of the steering vectors of two orthogonal uniform linear arrays:

(1) $$a\left(\theta ,\phi \right)=a_{y}\left(\theta ,\phi \right)⊗a_{x}\left(\theta ,\phi \right)\in C^{N\times 1},$$

where ⊗ denotes the Kronecker product, and the vectors $a_{x}\left(\theta ,\phi \right)$ and $a_{y}\left(\theta ,\phi \right)$ are defined as

(2) $$a_{x}\left(\theta ,\phi \right)={\left[1,e^{-j2\pi \frac{d}{\lambda }u},\ldots ,e^{-j2\pi \frac{d}{\lambda }\left(N_{x}-1\right)u}\right]}^{T},$$

(3) $$a_{y}\left(\theta ,\phi \right)={\left[1,e^{-j2\pi \frac{d}{\lambda }v},\ldots ,e^{-j2\pi \frac{d}{\lambda }\left(N_{y}-1\right)v}\right]}^{T}.$$

Here, the spatial frequencies in the *x* and *y* directions are respectively defined as u=sinθcosϕ and v=sinθsinϕ.

At discrete time index *t*, the baseband equivalent received signal vector at the satellite antenna array can be modeled as the superposition of the desired signal, multiple interference signals, and additive noise:

(4) $$x\left(t\right)=s\left(t\right)a_{s}+\sum_{k=1}^{K}i_{k}\left(t\right)a_{k}+n\left(t\right),\;t=1,2,\ldots ,T,$$

where s(t) denotes the desired signal waveform transmitted from the legitimate ground terminal located at direction $\left(\theta_{s},\phi_{s}\right)$, and $a_{s}=a\left(\theta_{s},\phi_{s}\right)$ is the corresponding desired steering vector. The term $i_{k}\left(t\right)$ denotes the *k*-th interference signal originating from a terrestrial 5G base station located at direction $\left(\theta_{k},\phi_{k}\right)$, with its steering vector denoted as $a_{k}=a\left(\theta_{k},\phi_{k}\right)$. The variable *K* represents the total number of active interference sources, and *T* is the number of available data snapshots. The vector $n\left(t\right)\in C^{N\times 1}$ represents the additive white Gaussian noise (AWGN) at the receiver, modeled as a circularly symmetric complex Gaussian random vector, i.e., $n\left(t\right)∼\mathrm{CN}\left(0,\sigma_{n}^{2}I_{N}\right)$.

We assume that the desired signal, the interference signals, and the noise are mutually statistically uncorrelated and follow zero-mean circularly symmetric complex Gaussian distributions. The average power of the desired signal is defined as $\sigma_{s}^{2}={E\{|s\left(t\right)|}^{2}\}$, and the average power of the *k*-th interference signal is defined as $\sigma_{i,k}^{2}=E\{|i_{k}\left(t\right){|}^{2}\}$ for k=1,…,K.

Under these statistical assumptions, the theoretical covariance matrix of the received signal vector is given by

(5) $$R=E\left\{x\left(t\right)x^{H}\left(t\right)\right\}=R_{s}+R_{i}+R_{n},$$

where the desired signal covariance matrix is $R_{s}=\sigma_{s}^{2}a_{s}a_{s}^{H}$, the interference covariance matrix is $R_{i}=\sum_{k=1}^{K}\sigma_{i,k}^{2}a_{k}a_{k}^{H}=A_{i}\Sigma_{i}A_{i}^{H}$, and the noise covariance matrix is $R_{n}=\sigma_{n}^{2}I_{N}$. Here, $A_{i}=\left[a_{1},a_{2},\ldots ,a_{K}\right]\in C^{N\times K}$ is the interference steering matrix, and $\Sigma_{i}=\mathrm{diag}\left(\sigma_{i,1}^{2},\sigma_{i,2}^{2},\ldots ,\sigma_{i,K}^{2}\right)$ is the diagonal matrix containing the true interference powers. The interference-plus-noise covariance matrix (INCM), which is the central component for optimal beamforming design, is defined as

(6) $$R_{i+n}=R_{i}+R_{n}=\sum_{k=1}^{K}\sigma_{i,k}^{2}a_{k}a_{k}^{H}+\sigma_{n}^{2}I_{N}.$$

In practical scenarios, the theoretical covariance matrix R is unavailable and must be approximated using the finite received data. The sample covariance matrix (SCM) is computed as the time average over the *T* snapshots:

(7) $$\hat{R}=\frac{1}{T}\sum_{t=1}^{T}x\left(t\right)x^{H}\left(t\right)=\frac{1}{T}XX^{H},$$

where $X=\left[x\left(1\right),x\left(2\right),\ldots ,x\left(T\right)\right]\in C^{N\times T}$ is the received data matrix.

**Figure 2:**
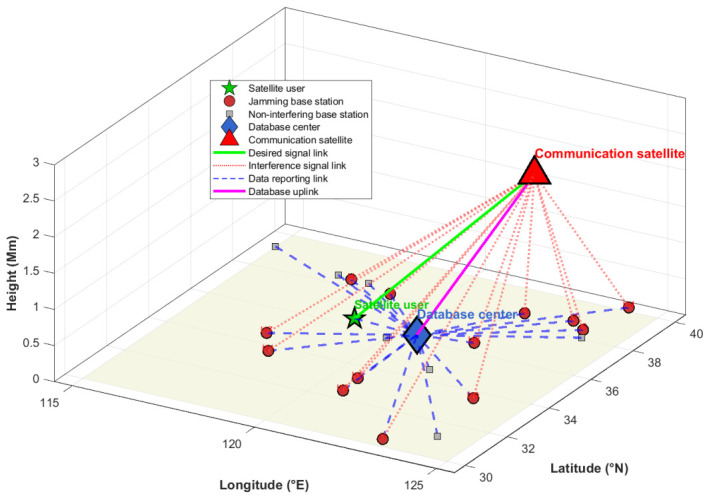
Database-assisted satellite communication system model.

A distinguishing feature of this work is the exploitation of coarse prior spatial information regarding potential interferers, provided by a terrestrial network database. As shown in Figure 2, emergency user terminals and terrestrial base stations report their geographical locations to a centralized spectrum database, which aggregates and forwards this information to the satellite. Utilizing the satellite’s orbital position and the reported terrestrial coordinates, the database computes and provides nominal angular directions ${\left\{\left({\hat{\theta }}_{k},{\hat{\phi }}_{k}\right)\right\}}_{k=1}^{K}$ for potential interferers. It is important to note that not all base stations listed in the database pose effective interference to the satellite link; only those transmitting in the overlapping frequency bands, possessing suitable elevation angles, and experiencing line-of-sight (LoS) propagation conditions will produce non-negligible interference. The proposed method is designed to identify and suppress these active components through spatial refinement and power recovery.

During the reception of the desired signal, the satellite may suffer from severe co-channel or adjacent-channel interference generated by this subset of active 5G base stations. By leveraging the location-based prior information provided by the database, the satellite can efficiently construct accurate beamforming weight vectors without resorting to blind global searches. As a result, a high-gain mainlobe is formed toward the intended user, while deep spatial nulls are steered precisely toward the interference directions, thereby suppressing the interference and ensuring the quality of service for legitimate satellite users.

**Figure 3:**
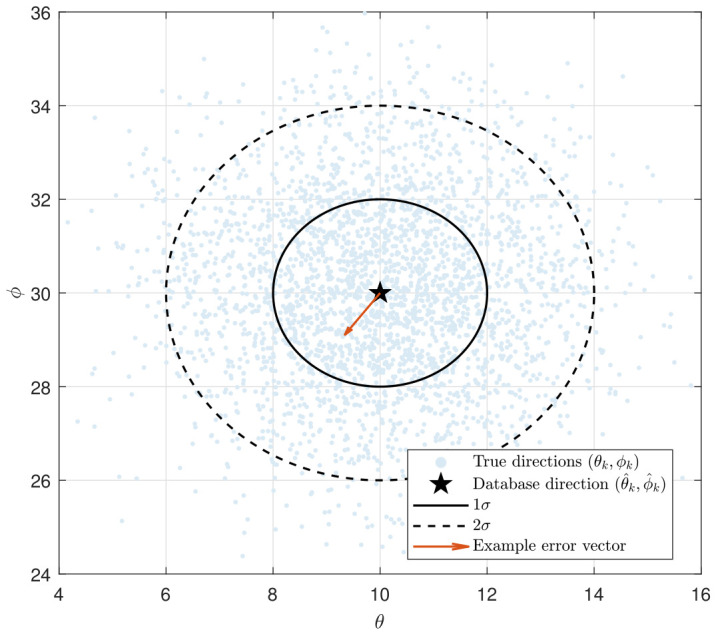
Illustration of the database angular error model in the azimuth–elevation domain.

However, the practical accuracy of the database coordinates is fundamentally limited by several error sources: satellite ephemeris perturbations, imprecise registration of ground base station locations, coordinate transformation quantization, and atmospheric refraction effects. To capture these uncertainties, the angular errors within the database are modeled as additive perturbations:

(8) $$\theta_{k}={\hat{\theta }}_{k}+\Delta \theta_{k},\;\phi_{k}={\hat{\phi }}_{k}+\Delta \phi_{k},$$

where $\left({\hat{\theta }}_{k},{\hat{\phi }}_{k}\right)$ denotes the nominal azimuth and elevation angles provided by the database, and $\left(\theta_{k},\phi_{k}\right)$ denotes the corresponding true angles of arrival. The perturbation terms $\Delta \theta_{k}$ and $\Delta \phi_{k}$ are modeled as independent zero-mean Gaussian random variables with standard deviation $\sigma_{\mathrm{db}}$, which quantifies the statistical pointing error of the database. Figure 3 illustrates this angular-error model in the (θ,ϕ) plane: the star marker indicates the nominal database direction $\left({\hat{\theta }}_{k},{\hat{\phi }}_{k}\right)$, the scatter points represent Monte Carlo realizations of the actual direction $\left(\theta_{k},\phi_{k}\right)$, and the solid/dashed contours depict the $1\sigma_{\mathrm{db}}$ and $2\sigma_{\mathrm{db}}$ error boundaries, respectively. The arrow shows a specific realization of the error vector $\left(\Delta \theta_{k},\Delta \phi_{k}\right)$.

The ultimate objective of the satellite receiver is to design a linear beamforming weight vector $w\in C^{N\times 1}$ that maximizes the output SINR, defined as:

(9) $$\mathrm{SINR}=\frac{\sigma_{s}^{2}{\left|w^{H}a_{s}\right|}^{2}}{w^{H}R_{i+n}w}.$$

When the true INCM $R_{i+n}$ is perfectly known, the optimal solution is given by the MVDR beamformer:

(10) $$w_{\mathrm{opt}}=\frac{R_{i+n}^{-1}a_{s}}{a_{s}^{H}R_{i+n}^{-1}a_{s}}.$$

However, obtaining an accurate estimate of $R_{i+n}$ in practical LEO emergency scenarios is highly challenging due to three compounding factors:

- Snapshot Starvation: The number of available stationary snapshots *T* is typically small relative to the large array dimension *N*, rendering the SCM ill-conditioned and causing severe subspace leakage.
- Signal Contamination: The desired signal component is embedded within the received data X. Directly using the SCM $\hat{R}$ in place of $R_{i+n}$ leads to severe signal self-nulling, especially at high SNR.
- Prior Uncertainty: While the database provides structural prior information to bypass the snapshot limitation, the inevitable angular mismatch ($\sigma_{\mathrm{db}}>0$) causes conventional database-assisted nulls to miss the true interferers, leading to catastrophic interference leakage.

The proposed algorithm, detailed in the next section, is specifically designed to overcome these three challenges simultaneously.

## 3. Proposed Database-Aided Robust Beamforming Algorithm

To overcome the limitations of purely data-driven methods under snapshot starvation and the vulnerability of conventional database-assisted methods to angular mismatch, we propose a robust framework that intelligently integrates database priors with data-driven spatial spectrum estimation. The core philosophy of the proposed method is to decouple the interference subspace estimation into two sequential tasks: localized spatial refinement and iterative power reconstruction. This decoupling avoids the prohibitive computational complexity and false-peak susceptibility associated with global 2D angular searches.

From an intuitive perspective, the proposed algorithm can be interpreted as a progressive interference localization and reconstruction procedure. Instead of blindly estimating the entire spatial spectrum over the full angular domain, the database first provides coarse candidate regions where interferers are likely to exist. The local refinement stage then accurately calibrates the interference directions within these small neighborhoods, while the TAA stage selectively estimates only the dominant interference powers and suppresses weak spurious components.

The proposed framework consists of three stages. First, a database-guided local refinement procedure searches for high-resolution Capon spectrum peaks strictly within a constrained neighborhood around each nominal database direction. This step corrects the database angular mismatch while explicitly protecting the desired signal. Second, a truncated adaptive amplitude (TAA) estimation procedure iteratively recovers the true interference powers along the refined directions. By incorporating an adaptive truncation mechanism, this step effectively prunes inactive interferers and suppresses noise-induced spurious components. Finally, an accurate INCM is reconstructed using the refined directions and powers, from which the optimal MVDR beamformer weights are computed.

### 3.1. Database-Guided Local Refinement

The raw angular coordinates provided by the terrestrial database, denoted as ${\left\{\left({\hat{\theta }}_{k},{\hat{\phi }}_{k}\right)\right\}}_{k=1}^{K}$, serve as coarse initial guesses for the interference locations. To compensate for the inevitable database pointing errors modeled in (7), a local spatial search is performed to pinpoint the exact interference directions. We employ the Capon spatial spectrum for this refinement due to its superior resolution and its inherent ability to suppress interference from other directions while evaluating a specific look direction.

For the *k*-th database direction, the local search region $S_{k}$ is defined as a rectangular angular window centered at the nominal coordinates:

(11) $$S_{k}=\{\left(\theta ,\phi \right):|\theta -{\hat{\theta }}_{k}|\leq \Delta_{s},\;|\phi -{\hat{\phi }}_{k}\left|\leq \Delta_{s}\right\},$$

where $\Delta_{s}$ is the search window half-width. To ensure that the true interferer is captured, $\Delta_{s}$ should be chosen proportionally to the statistical standard deviation of the database error (e.g., $\Delta_{s}\approx 2\sigma_{\mathrm{db}}$ to $3\sigma_{\mathrm{db}}$).

A critical issue in spatial refinement is the risk of signal self-nulling: if an interference search window happens to overlap with the mainlobe of the desired signal, the refinement process might erroneously lock onto the desired signal, leading to catastrophic signal cancellation in the subsequent beamforming stage. To strictly prevent this, we define a circular exclusion zone around the known desired signal direction $\left(\theta_{s},\phi_{s}\right)$:

(12) $$B_{s}=\left\{\left(\theta ,\phi \right):\sqrt{{\left(\theta -\theta_{s}\right)}^{2}+{\left(\phi -\phi_{s}\right)}^{2}}<\delta_{\mathrm{exc}}\right\},$$

where $\delta_{\mathrm{exc}}$ is the exclusion radius, typically set to the half-power beamwidth of the array. The refined interference direction is then obtained by maximizing the Capon spectrum strictly within the valid region $S_{k}\setminus B_{s}$:

(13) $$\left({\tilde{\theta }}_{k},{\tilde{\phi }}_{k}\right)=\arg\max_{\left(\theta ,\phi \right)\in S_{k}\setminus B_{s}}\frac{1}{a{\left(\theta ,\phi \right)}^{H}{\hat{R}}^{-1}a\left(\theta ,\phi \right)}.$$

This constrained local search ensures that the refined directions accurately align with the true interferers without compromising the desired signal integrity.

### 3.2. Truncated Adaptive Amplitude Estimation

Although the Capon spectrum provides high-resolution direction estimates, its corresponding power estimates are known to be biased downward, particularly in snapshot-limited regimes or when interference sources are closely spaced. To accurately reconstruct the INCM, an unbiased estimation of the interference powers is required. We develop a Truncated Adaptive Amplitude (TAA) estimation procedure that iteratively refines the power estimates using a weighted least squares criterion, operating exclusively on the refined spatial basis.

Given the refined directions $\left\{\left({\tilde{\theta }}_{k},{\tilde{\phi }}_{k}\right)\right\}$, we construct the refined interference steering matrix:

(14) $$\tilde{A}=\left[{\tilde{a}}_{1},{\tilde{a}}_{2},\ldots ,{\tilde{a}}_{K}\right],\;{\tilde{a}}_{k}=a\left({\tilde{\theta }}_{k},{\tilde{\phi }}_{k}\right).$$

As an initialization, the interference powers are approximated using a standard Capon estimator:

(15) $$P_{k}^{\left(0\right)}=\frac{1}{{\tilde{a}}_{k}^{H}{\left(\hat{R}+\epsilon I_{N}\right)}^{-1}{\tilde{a}}_{k}},\;k=1,\ldots ,K,$$

where ϵ>0 is a small diagonal loading factor added to ensure the invertibility of the sample covariance matrix $\hat{R}$ in severely snapshot-starved cases.

At the *ℓ*-th iteration ($\ell =1,2,\ldots ,L_{\max}$, where $L_{\max}$ is the maximum number of iterations), the structured covariance matrix is updated using the power estimates from the previous iteration:

(16) $$R^{\left(\ell \right)}=\tilde{A}\;\mathrm{diag}\left(P_{1}^{\left(\ell -1\right)},\ldots ,P_{K}^{\left(\ell -1\right)}\right)\;{\tilde{A}}^{H}+{\hat{\sigma }}_{n}^{2}I_{N}.$$

Here, ${\hat{\sigma }}_{n}^{2}$ represents the background noise power. Assuming that the signal-plus-interference subspace dimension is at most K+1, the noise power can be robustly estimated by averaging the eigenvalues corresponding to the noise subspace of $\hat{R}$:

(17) $${\hat{\sigma }}_{n}^{2}=\frac{1}{N-K-1}\sum_{n=K+2}^{N}\lambda_{n},$$

where $\lambda_{n}$ are the eigenvalues of $\hat{R}$ sorted in descending order.

To update the power of the *k*-th interferer, we design an MVDR-like spatial filter $w_{k}^{\left(\ell \right)}={\left[R^{\left(\ell \right)}\right]}^{-1}{\tilde{a}}_{k}$ that passes the signal from ${\tilde{a}}_{k}$ without distortion while suppressing all other interferers based on the current covariance structure $R^{\left(\ell \right)}$. Applying this filter to the received data matrix $X\in C^{N\times T}$ yields the isolated interference signal output:

(18) $$z_{k}^{\left(\ell \right)}={\left(w_{k}^{\left(\ell \right)}\right)}^{H}X={\tilde{a}}_{k}^{H}{\left[R^{\left(\ell \right)}\right]}^{-1}X\in C^{1\times T}.$$

The spatial filter introduces a gain factor $d_{k}^{\left(\ell \right)}$ in the target direction, defined as:

(19) $$d_{k}^{\left(\ell \right)}={\tilde{a}}_{k}^{H}{\left[R^{\left(\ell \right)}\right]}^{-1}{\tilde{a}}_{k}.$$

By compensating for this gain and averaging over the *T* snapshots, the updated power estimate is derived as:

(20) $$\begin{array}{cc} P_{k}^{\left(\ell \right)} & =\frac{1}{T}\frac{∥z_{k}^{\left(\ell \right)}{∥}_{2}^{2}}{|d_{k}^{\left(\ell \right)}{|}^{2}}=\frac{1}{T}\frac{{∥{\tilde{a}}_{k}^{H}{\left[R^{\left(\ell \right)}\right]}^{-1}X∥}_{2}^{2}}{|{\tilde{a}}_{k}^{H}{\left[R^{\left(\ell \right)}\right]}^{-1}{\tilde{a}}_{k}{|}^{2}},\;k=1,\ldots ,K. \end{array}$$

This iterative formulation effectively minimizes the weighted least squares fitting error between the sample covariance and the structured covariance model.

Furthermore, the database may contain registered base stations that are currently inactive or geographically blocked, leading to false alarms. Alternatively, severe noise may generate spurious peaks during the local refinement stage. To enhance the sparsity of the spatial spectrum and eliminate these phantom sources, a hard truncation operation is applied at the end of each iteration:

(21) $$P_{k}^{\left(\ell \right)}\leftarrow \left\{\begin{array}{cc} P_{k}^{\left(\ell \right)}, & \mathrm{if}\;P_{k}^{\left(\ell \right)}\geq \tau \\ 0, & \mathrm{otherwise} \end{array}\right..$$

The truncation threshold τ is adaptively determined based on both the dominant interference power and the noise floor:

(22) $$\tau =\max\left(\eta \;\cdot \;\max_{k}P_{k}^{\left(\ell \right)},\;\frac{1}{2}{\hat{\sigma }}_{n}^{2}\right),$$

where η is a relative threshold factor (e.g., $10^{-3}$). This ensures that any estimated power falling below the noise floor or significantly weaker than the dominant interferers is discarded. The iteration continues until the relative change in the power vector $P^{\left(\ell \right)}={\left[P_{1}^{\left(\ell \right)},\ldots ,P_{K}^{\left(\ell \right)}\right]}^{T}$ satisfies the convergence criterion:

(23) $$\frac{∥P^{\left(\ell \right)}-P^{\left(\ell -1\right)}{∥}_{2}}{∥P^{\left(\ell \right)}{∥}_{2}}<\epsilon_{\mathrm{tol}},$$

where $\epsilon_{\mathrm{tol}}$ is a predefined tolerance (e.g., $10^{-3}$).

Intuitively, the TAA procedure can be viewed as an interference power refinement mechanism operating on a reduced spatial basis. At each iteration, the structured covariance matrix suppresses cross-coupling among interferers, allowing the spatial filter to isolate the contribution of each refined direction more accurately. The truncation operation further removes weak or noise-dominated components, thereby gradually stabilizing the covariance reconstruction and improving robustness under limited snapshots.

### 3.3. INCM Reconstruction and Beamformer Computation

Once the TAA algorithm converges, we obtain a highly reliable set of active interference directions and their corresponding powers. Crucially, because this estimation process explicitly excludes the desired signal region $B_{s}$, the resulting components represent a pure interference-plus-noise profile. The INCM is then analytically reconstructed as:

(24) $${\hat{R}}_{i+n}=\sum_{k=1}^{K}\alpha_{k}P_{k}\;{\tilde{a}}_{k}{\tilde{a}}_{k}^{H}+{\hat{\sigma }}_{n}^{2}I_{N}+\gamma_{\mathrm{DL}}I_{N}.$$

In this formulation, two important robustness enhancements are introduced. First, the scaling factor $\alpha_{k}\geq 1$ acts as a conservative interference margin. Although the local refinement stage significantly corrects the database errors, minor residual mismatches or slight angular spread may still exist. Setting $\alpha_{k}>1$ (typically $\alpha_{k}=2$) artificially inflates the interference power in the reconstructed matrix, which forces the subsequent beamformer to synthesize a slightly wider and deeper spatial null around ${\tilde{a}}_{k}$, thereby improving robustness against residual errors. Second, $\gamma_{\mathrm{DL}}$ is a diagonal loading term (typically $\gamma_{\mathrm{DL}}=2{\hat{\sigma }}_{n}^{2}$) designed to stabilize the noise subspace and ensure the reconstructed INCM is well-conditioned for inversion, which is particularly beneficial in extreme snapshot-starved scenarios.

Finally, substituting the reconstructed INCM into the theoretical MVDR formulation yields the proposed robust beamforming weight vector:

(25) $$w_{\mathrm{prop}}=\frac{{\hat{R}}_{i+n}^{-1}a_{s}}{a_{s}^{H}{\hat{R}}_{i+n}^{-1}a_{s}}.$$

The final enhanced output signal of the antenna array is obtained by applying the weight vector to the received data:

(26) $$y\left(t\right)=w_{\mathrm{prop}}^{H}x\left(t\right).$$

The complete proposed algorithm is summarized in Algorithm 1. In practice, the parameters of the proposed framework are selected according to physically interpretable principles rather than exhaustive tuning. Specifically, the local search radius $\Delta_{s}$ is chosen proportional to the expected database uncertainty, while the truncation factor η controls the minimum retained interference level relative to the dominant interferer.
**Algorithm 1** Proposed Database-aided Robust Beamforming**Require:** 
Sample covariance matrix $\hat{R}$, data matrix X, database directions ${\left\{\left({\hat{\theta }}_{k},{\hat{\phi }}_{k}\right)\right\}}_{k=1}^{K}$, desired signal steering vector $a_{s}$**Ensure:** 
Beamformer weight vector $w_{\mathrm{prop}}$  1:** Database-guided direction refinement:**  2: **for** k=1 to *K* **do**  3:      Refine direction by local Capon spectrum maximization$$\left({\tilde{\theta }}_{k},{\tilde{\phi }}_{k}\right)=\arg\max_{\left(\theta ,\phi \right)\in S_{k}\setminus B_{s}}\frac{1}{a^{H}\left(\theta ,\phi \right){\hat{R}}^{-1}a\left(\theta ,\phi \right)}$$  4: **end for**  5: **Truncated adaptive amplitude estimation:**  6: Initialize $P_{k}^{\left(0\right)}$ using Capon estimator  7: **for** ℓ=1 to $L_{\max}$ **do**  8:      Construct $R^{\left(\ell \right)}=\tilde{A}\mathrm{diag}\left(P^{\left(\ell -1\right)}\right){\tilde{A}}^{H}+{\hat{\sigma }}_{n}^{2}I$  9:      **for** k=1 to *K* **do**10:          Update power$$P_{k}^{\left(\ell \right)}=\frac{1}{T}\frac{{∥{\tilde{a}}_{k}^{H}{\left[R^{\left(\ell \right)}\right]}^{-1}X∥}_{2}^{2}}{|{\tilde{a}}_{k}^{H}{\left[R^{\left(\ell \right)}\right]}^{-1}{\tilde{a}}_{k}{|}^{2}}$$11:      **end for**12:      Apply truncation and check convergence13: **end for**14: **INCM reconstruction and beamforming:**15: Reconstruct ${\hat{R}}_{i+n}$ using refined directions and powers16: Compute MVDR beamformer$$w_{\mathrm{prop}}=\frac{{\hat{R}}_{i+n}^{-1}a_{s}}{a_{s}^{H}{\hat{R}}_{i+n}^{-1}a_{s}}$$17: **return** $w_{\mathrm{prop}}$

The parameters $\alpha_{k}$ and $\gamma_{\mathrm{DL}}$ also play important roles in the stability of the proposed framework. The weighting coefficients $\alpha_{k}$ regulate the contribution of each refined direction in the covariance reconstruction, preventing dominance of spurious components. The diagonal loading factor $\gamma_{\mathrm{DL}}$ improves robustness under limited snapshots by stabilizing the covariance inversion. In practice, moderate values of these parameters provide a balance between robustness and estimation accuracy.

## 4. Computational Complexity Analysis

Let *N* denote the total number of array elements, *T* the number of available snapshots, *K* the number of database-provided interference directions, *L* the number of iterations required for the truncated adaptive amplitude (TAA) estimation to converge, and *G* the number of grid points evaluated in the local Capon spectrum refinement for each database direction. By leveraging matrix factorization and linear equation solvers instead of explicit matrix inversion, the dominant computational costs of the proposed algorithm can be analyzed as follows.

The proposed methodology fundamentally consists of database-guided local direction refinement, TAA-based power estimation, and INCM reconstruction. Computing the sample covariance matrix requires $O(N^{2}T)$ operations, and its subsequent Cholesky factorization requires $O(N^{3})$ operations. In the local refinement stage, the Capon spectrum is evaluated over *G* localized grid points for each of the *K* database directions. Each evaluation involves one forward-backward substitution (linear solve) and an inner product, resulting in a complexity of $O(KGN^{2})$.

In the TAA stage, each of the *L* iterations requires constructing the structured covariance matrix and performing one matrix factorization with a complexity of $O(N^{3})$. Subsequently, solving the linear system $R^{\left(\ell \right)}\setminus X$ to isolate the interference signals requires $O(N^{2}T)$ operations. Updating the *K* interference powers incurs an additional cost on the order of $O(N^{2}K)$. Therefore, the overall computational complexity of the proposed algorithm is dominated by:

(27) $$O\;\left(LN^{3}+LN^{2}T+KGN^{2}\right).$$

In snapshot-limited scenarios (where T≪N), the $O(LN^{3})$ term is the dominant factor, whereas for larger *T*, the $O(LN^{2}T)$ term becomes the primary contributor. It is important to note that the adaptive truncation operation in the TAA stage effectively prunes inactive or spurious directions, dynamically reducing the active dimension *K* in later iterations and thereby lowering the practical computational burden.

For comparison, conventional DL-MVDR mainly involves diagonal loading followed by a single covariance matrix inversion, yielding a complexity primarily determined by $O(N^{3}+N^{2}T)$ [19]. The ESB-based method additionally requires the eigenvalue decomposition of the sample covariance matrix, which incurs a strict $O(N^{3})$ cost regardless of the snapshot count [24]. Robust DB-based INCM reconstruction methods rely on summations of rank-one covariance components and a final matrix inversion, with complexity related to the number of database directions and the array size. Crucially, blind IAA-based beamforming typically involves significantly higher complexity, $O(L_{\mathrm{IAA}}N^{3}+L_{\mathrm{IAA}}G_{\mathrm{global}}N^{2})$, due to its reliance on a dense global angular grid $G_{\mathrm{global}}$ (where $G_{\mathrm{global}}\gg KG$) [26].

For the default simulation setting with N=64, K=25, and T=100, the onboard storage requirement is modest. Using double-precision complex numbers, the received data matrix X requires about NT×16≈102.4 kB, each 64×64 covariance matrix requires about 65.5 kB, and the refined steering matrix requires about NK×16≈25.6 kB. Even when several intermediate covariance matrices and Cholesky factors are stored simultaneously, the total working memory remains below 1 MB. Therefore, the proposed algorithm does not impose a heavy onboard memory burden. For latency estimation, consider a typical local grid resolution of 0.5° within the default search half-width $\Delta_{s}=4{}^{\circ}$, which gives approximately G=17×17=289 local grid points per database direction. With L=8 TAA iterations, the dominant operation count is approximately $LN^{3}+LN^{2}T+KGN^{2}\approx 3.5\times 10^{7}$ complex arithmetic operations. This workload is mainly composed of matrix factorization, linear solves, and independent Capon spectrum evaluations over local angular windows. These operations are highly parallelizable on DSP/FPGA platforms. Since the proposed method avoids a dense global two-dimensional angular search, its latency is expected to be dominated by a small number of structured matrix operations and local spectrum evaluations, making real-time or near-real-time onboard execution feasible for snapshot-block processing.

The power consumption depends on the specific onboard processor, numerical precision, clock rate, and implementation architecture. However, the proposed method reduces power demand at the algorithmic level by restricting spectrum evaluation to localized database-guided regions and by pruning inactive directions through truncation. Thus, compared with blind global-spectrum methods such as IAA, the proposed algorithm offers a more favorable trade-off among computational cost, memory footprint, latency, and robustness for practical LEO payload implementation.

## 5. Simulation Results

In this section, Monte Carlo simulations are conducted to evaluate the performance of the proposed database-aided robust beamforming algorithm. Unless otherwise specified, the simulation parameters are summarized in Table 1. An 8×8 UPA with half-wavelength inter-element spacing is considered. The setup models a satellite-to-ground communication scenario with limited snapshot support, which is a typical practical constraint. To emulate a more realistic scenario, the elevation angles of all signals are constrained to θ∈[0°–30°].

The desired signal-to-noise ratio (SNR) is set to 0 dB, the average interference-to-noise ratio (INR) is set to 25 dB, and the number of snapshots is fixed at T=100. The interferer directions are randomly generated with a minimum angular separation from the desired signal to avoid overlap. Database angular mismatches are modeled as independent Gaussian perturbations with a standard deviation $\sigma_{\mathrm{db}}$ of 2°.

The adopted simulation setting is intended to isolate the core behavior of the proposed database-aided refinement and covariance reconstruction mechanism. In practical satellite-to-ground communication systems, effects such as correlated interference, array calibration errors, and steering-vector perturbations may further introduce covariance mismatch and directional uncertainty. These practical imperfections are partially reflected by the database angular mismatch model and the limited-snapshot setting considered in the simulations. Moreover, the proposed local refinement and conservative covariance reconstruction mechanisms are designed to improve robustness against such uncertainty.

**Figure 4:**
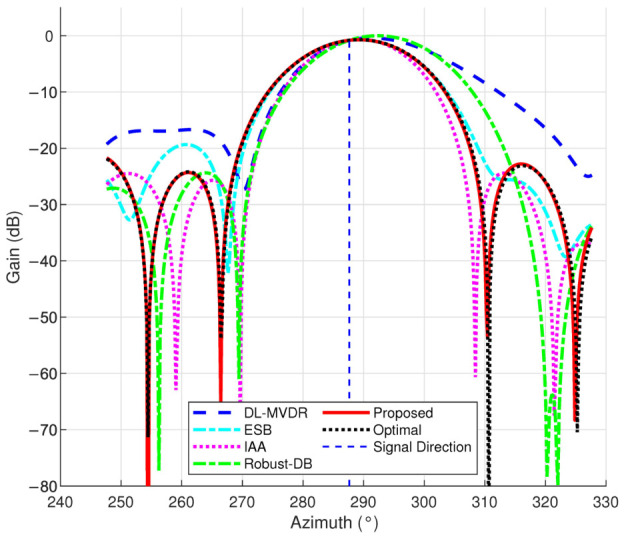
Output beampatterns of different methods.

Figure 4 presents the output beampatterns of different methods. For visualization, the planar array response is shown as an azimuth cut at the elevation angle of the desired signal direction. The desired signal is randomly generated, while multiple interferers impinge from other directions.

The proposed method forms a sharp and well-focused mainlobe toward the desired direction and places deep and accurate nulls at the true interference locations. In contrast, DL-MVDR [19], ESB [24], and IAA [26] exhibit distorted mainlobes and insufficient null depths. This degradation is mainly caused by inaccurate covariance estimation under limited snapshots, leading to subspace leakage and imprecise interference suppression. Although Robust-DB [32] utilizes database information, its performance degrades under database mismatch, as it places nulls at erroneous directions. This results in residual interference leakage and a widened mainlobe. Overall, the results demonstrate that mismatch-aware local refinement enables more accurate spatial filtering.

**Figure 5:**
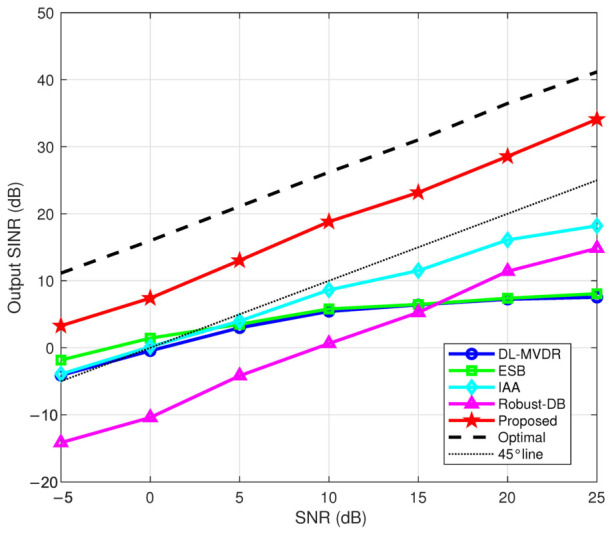
Output SINR versus SNR for different methods.

Figure 5 shows the output SINR versus the input SNR with INR fixed at 25 dB. As the SNR increases, all methods improve due to enhanced signal quality.

The proposed method consistently achieves the highest output SINR across the entire SNR range and closely approaches the optimal benchmark. Its advantage is particularly evident in the low-SNR region, where noise and limited snapshots make covariance estimation unreliable for DL-MVDR, ESB, and IAA, leading to signal cancellation and degraded interference suppression. Robust-DB benefits from database guidance but still shows a noticeable performance gap due to mismatch effects. These results indicate that accurate mismatch compensation and covariance reconstruction are critical for achieving robust performance in low-SNR conditions.

**Figure 6:**
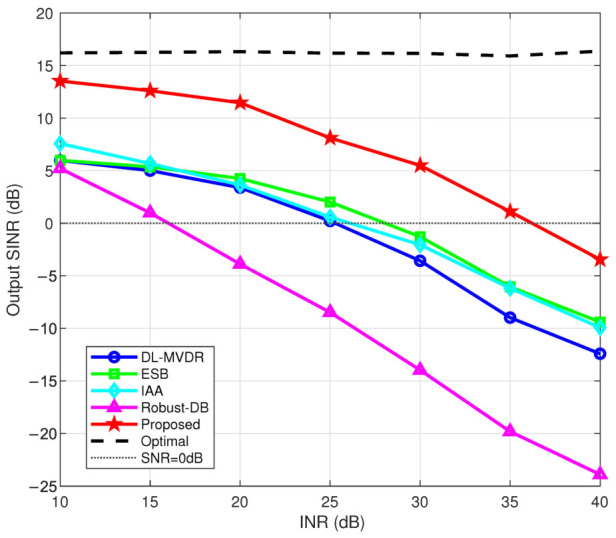
Output SINR versus INR for different methods.

Figure 6 depicts the output SINR as a function of the INR at a fixed input SNR of 0 dB. As the interference power increases, the performance of all methods declines due to the increasing dominance of interference components.

DL-MVDR, ESB, and IAA degrade rapidly because they rely on accurate covariance estimation, which becomes unreliable under strong interference. In contrast, the proposed method exhibits a much slower performance decline and maintains stable SINR across a wide INR range. This robustness is attributed to the local refinement stage, which accurately calibrates interference directions and ensures precise null placement. Robust-DB also suffers significant degradation under high INR, highlighting its sensitivity to database mismatch. These results confirm the importance of mismatch-aware processing in strong interference scenarios.

**Figure 7:**
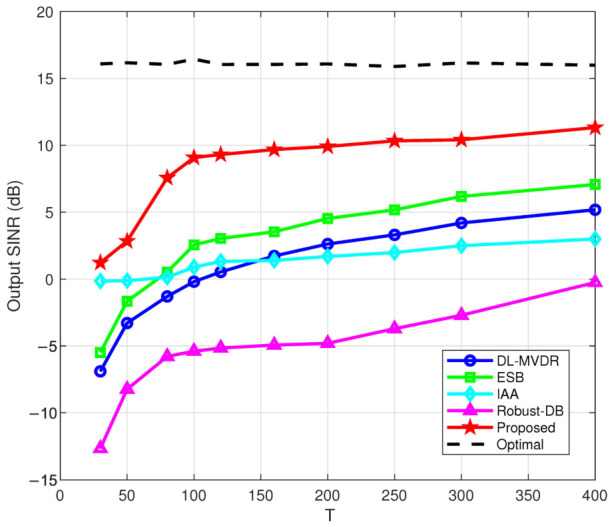
Output SINR versus snapshots for different methods.

Figure 7 illustrates the output SINR as a function of the number of snapshots *T*. As expected, increasing *T* improves performance for all methods due to more accurate covariance estimation.

The proposed method significantly outperforms competing algorithms under limited snapshots, demonstrating strong sample efficiency. This is because the database-aided design reduces reliance on large sample support and enables accurate covariance reconstruction even in snapshot-starved conditions. In contrast, DL-MVDR and ESB suffer from severe performance degradation when *T* is small due to ill-conditioned covariance matrices, while IAA shows limited stability. Robust-DB remains constrained by database mismatch, resulting in a persistent performance gap. As *T* increases, all methods improve, but the proposed method converges faster and maintains a clear advantage.

**Figure 8:**
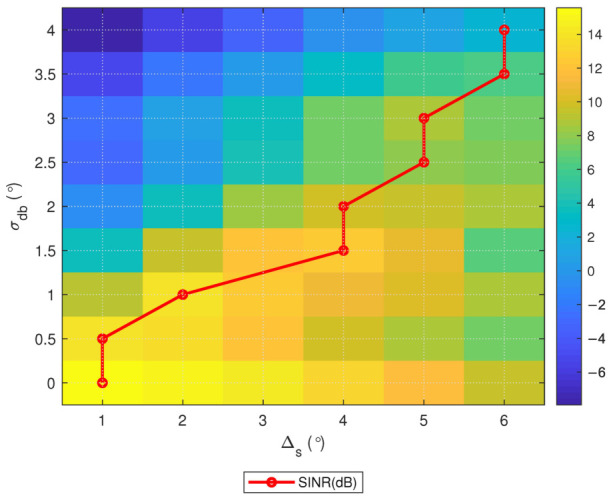
Output SINR versus database error deviation $\sigma_{\mathrm{db}}$ and local search radius $\Delta_{s}$.

Figure 8 shows the output SINR as a function of the database error $\sigma_{\mathrm{db}}$ and the local search radius $\Delta_{s}$. The results illustrate the robustness of the proposed local refinement mechanism and highlight the importance of proper parameter selection.

The search radius $\Delta_{s}$ should be large enough to cover the true interference direction under database uncertainty, but not too large to include noise or sidelobe artifacts. The results indicate that optimal performance is achieved when $\Delta_{s}$ scales with $\sigma_{\mathrm{db}}$. If $\Delta_{s}$ is too small, the refinement may miss the true interference direction, leading to inaccurate null placement. If it is too large, the inclusion of spurious components degrades performance. This provides a practical guideline: $\Delta_{s}$ should be selected according to the expected database uncertainty.

Overall, the simulation results confirm that the proposed method achieves robust interference suppression under SNR variation, strong interference, limited snapshots, and database angular mismatch. Compared with data-driven and conventional database-assisted baselines, its main advantage comes from the combination of database-guided local refinement and TAA-based covariance reconstruction. The parameter analysis further shows that the local search radius should be selected according to the expected database uncertainty. These results verify the effectiveness of the proposed framework.

## 6. Conclusions

This paper proposed a robust database-aided beamforming algorithm designed to effectively suppress uplink terrestrial interference in low Earth orbit (LEO) satellite emergency communications. Recognizing the limitations of conventional adaptive beamformers in snapshot-deficient environments, the proposed algorithm strategically leverages prior interference directional information from an external database. To overcome the inevitable inaccuracies inherent in such databases, a database-guided local refinement procedure is first introduced, which accurately calibrates the interference directions by performing a localized Capon spectrum search while strictly protecting the desired signal region. Subsequently, a truncated adaptive amplitude (TAA) estimation method is developed to iteratively estimate the interference powers. By incorporating an adaptive truncation mechanism, this step effectively eliminates spurious peaks and noise-induced artifacts, ensuring a highly reliable power estimation. Finally, a precise interference-plus-noise covariance matrix (INCM) is reconstructed to compute the optimal beamformer weights. Comprehensive simulation results demonstrate that the proposed method forms highly accurate mainlobes and deep nulls. It consistently achieves an output SINR that closely approaches the theoretical optimum, significantly outperforming existing benchmarks under severe practical constraints, including limited snapshots, strong interference, and significant database angular mismatches. The study also provides a practical guideline for parameter selection, confirming the robustness and suitability of the proposed algorithm for real-world satellite-to-ground emergency communication scenarios where reliable links are critical. Future work will focus on providing a rigorous theoretical analysis of the proposed framework, including convergence behavior of the TAA iterations, statistical estimation properties, and robustness bounds under database mismatch.

## Figures and Tables

**Table 1:** The Parameters of the Simulation.

Parameter	Value
UPA size ($N_{x}\times N_{y}$)	8×8
Inter-element spacing *d*	λ/2
Input SNR	0 dB
Average INR	25 dB
Number of interferers *K*	25
Database error Standard deviation $\sigma_{\mathrm{db}}$	2°
Local search half-width $\Delta_{s}$	4°
Snapshots *T*	100
Desired-signal exclusion radius $\delta_{\mathrm{exc}}$	4°
Elevation range of database-listed base stations	θ∈[0°,30°]

## Data Availability

The original contributions presented in this study are included in the article. Further inquiries can be directed to the corresponding author.
